# Nigral transcriptomic profiles in Engrailed-1 hemizygous mouse models of Parkinson’s disease reveal upregulation of oxidative phosphorylation-related genes associated with delayed dopaminergic neurodegeneration

**DOI:** 10.3389/fnagi.2024.1337365

**Published:** 2024-02-05

**Authors:** Lautaro Francisco Belfiori, Alfredo Dueñas Rey, Dorottya Mária Ralbovszki, Itzia Jimenez-Ferrer, Filip Fredlund, Sagar Shivayogi Balikai, Dag Ahrén, Kajsa Atterling Brolin, Maria Swanberg

**Affiliations:** ^1^Translational Neurogenetics Unit, Wallenberg Neuroscience Center, Department of Experimental Medical Sciences, Lund University, Lund, Sweden; ^2^Department of Biomolecular Medicine, Ghent University, Ghent, Belgium; ^3^Center for Medical Genetics, Ghent University Hospital, Ghent, Belgium; ^4^Department of Biology, National Bioinformatics Infrastructure Sweden (NBIS), SciLifeLab, Stockholm, Sweden

**Keywords:** Parkinson’s disease, substantia nigra, neurodegeneration, transcriptomics, genetic susceptibility, mitochondria

## Abstract

**Introduction:**

Parkinson’s disease (PD) is the second most common neurodegenerative disorder, increasing both in terms of prevalence and incidence. To date, only symptomatic treatment is available, highlighting the need to increase knowledge on disease etiology in order to develop new therapeutic strategies. Hemizygosity for the gene Engrailed-1 (*En1*), encoding a conserved transcription factor essential for the programming, survival, and maintenance of midbrain dopaminergic neurons, leads to progressive nigrostriatal degeneration, motor impairment and depressive-like behavior in SwissOF1 (OF1*-En1*^+/−^). The neurodegenerative phenotype is, however, absent in C57Bl/6j (C57*-En1*^+/−^) mice. *En1*^+/−^ mice are thus highly relevant tools to identify genetic factors underlying PD susceptibility.

**Methods:**

Transcriptome profiles were defined by RNAseq in microdissected substantia nigra from 1-week old OF1, OF1- *En1*^+/−^, C57 and C57- *En1*^+/−^ male mice. Differentially expressed genes (DEGs) were analyzed for functional enrichment. Neurodegeneration was assessed in 4- and 16-week old mice by histology.

**Results:**

Nigrostriatal neurodegeneration was manifested in OF1- *En1*^+/−^ mice by increased dopaminergic striatal axonal swellings from 4 to 16 weeks and decreased number of dopaminergic neurons in the SNpc at 16 weeks compared to OF1. In contrast, C57- *En1*^+/−^ mice had no significant increase in axonal swellings or cell loss in SNpc at 16 weeks. Transcriptomic analyses identified 198 DEGs between OF1- *En1*^+/−^ and OF1 mice but only 52 DEGs between C57- *En1*^+/−^ and C57 mice. Enrichment analysis of DEGs revealed that the neuroprotective phenotype of C57- *En1*^+/−^ mice was associated with a higher expression of oxidative phosphorylation-related genes compared to both C57 and OF1- *En1*^+/−^ mice.

**Discussion:**

Our results suggest that increased expression of genes encoding mitochondrial proteins before the onset of neurodegeneration is associated with increased resistance to PD-like nigrostriatal neurodegeneration. This highlights the importance of genetic background in PD models, how different strains can be used to model clinical and sub-clinical pathologies and provides insights to gene expression mechanisms associated with PD susceptibility and progression.

## 1 Introduction

Parkinson’s disease (PD) represents the fastest-growing neurodegenerative disease (Dorsey et al., 2018). PD is characterized by the loss of dopaminergic neurons in the substantia nigra pars compacta (SNpc), neuroinflammation and intracellular aggregates of α-Synuclein (α-Syn) in the form of Lewy bodies and neurites. The symptoms are progressive and include rigidity, bradykinesia and tremor, and non-motor symptoms such as anosmia, constipation, depression and sleep disorders. Current treatment options temporarily alleviate motor symptoms but do not target the underlying pathology (Poewe et al., 2017; Bloem et al., 2021).

A combination of genetic and pathophysiological evidence suggests mitochondrial malfunction (Błaszczyk, 2018), neuroinflammation (Grotemeyer et al., 2022), impairment of lipid metabolism (Galper et al., 2022), autophagy and dysfunctional unfolded protein response (Da Costa et al., 2020) to be part of PD etiology. The genetic component of PD has been extensively studied in familial cases with disease-causing mutations in, e.g., *SNCA*, *LRRK2*, and *PINK1*, and in case–control cohorts by genome-wide association (GWA) studies. *GBA1* is the most frequent genetic risk factor for PD due to variations conferring increased risk and mutations with a range from mild to severe effects (Parlar et al., 2023). *GBA1* encodes the lysosomal enzyme β-glucocerebrosidase (GCase) and homozygous mutation carriers develop the lysosomal storage disorder Gaucher disease. The functional link between GCase and PD can be attributed to impaired lysosomal function and endoplasmic reticulum stress leading to enhanced pathological modifications and reduced clearance of α-Syn, to impaired mitochondrial function impacting energy metabolism, and to activation of microglia contributing to neuroinflammation (Flores-Leon and Outeiro, 2023). There are thus clear links between genetics and neuropathological processes in PD, strongly supporting genetics as a tool to understand PD etiology. The so far largest PD GWA meta-analysis identified 90 risk loci that account for 16–36% of the heritable PD risk (Nalls et al., 2014, 2019). There is thus an important, yet unknown, genetic component that influences the incidence and progression of PD (Liu et al., 2021; Tan et al., 2021). To provide a biological context to interpret the results generated by genetic studies, large-scale gene expression (transcriptomic) approaches have been used in both murine models and human postmortem tissue (Hook et al., 2018; Dick et al., 2020; Monzón-Sandoval et al., 2020; Zaccaria et al., 2022). These studies have provided insight into gene expression changes in the SNpc associated with PD in an established and often advanced neurodegenerative context. We, however, lack biological insight into the processes underlying susceptibility to dopaminergic neurodegeneration and determining if, how and when PD will manifest.

Genetic animal models of PD have proven very useful in recapitulating key features of the disease (Konnova and Swanberg, 2018). One of them is the *Engrailed-1* hemizygous (*En1*^+/−^) mouse model, where one copy of *En1* is disrupted by a *LacZ* insertion (Saueressig et al., 1999). The EN1 protein is a highly conserved homeodomain transcription factor essential for the development and survival of the mesodiencephalic dopaminergic neurons, along with Engrailed-2 (EN2) and Pituitary homeobox 3 (PITX3) (Veenvliet et al., 2013). EN1 also has a role in the maintenance and sustainment of energy metabolism of dopaminergic neurons in adulthood (Rekaik et al., 2015a). Furthermore, SwissOF1- (OF1-) *En1*^+/−^ mice exhibit a spontaneous and thoroughly characterized PD-like neuropathology which recapitulates PD in terms of mitochondrial deficits, axonal degeneration, α-Syn-positive aggregates, diminished dopamine release in the dorsal striatum along with progressive loss of dopaminergic neurons in the SNpc and a more subtle neurodegenerative phenotype in the ventral tegmental area (VTA), (Sonnier et al., 2007; Alvarez-Fischer et al., 2011; Fuchs et al., 2012; Nordström et al., 2015; Rekaik et al., 2015b; Chatterjee et al., 2019).

We and others have previously shown that the consequences of *En1* hemizygosity in mice depend on genetic factors (Sgadò et al., 2006; Murcia et al., 2007; Sonnier et al., 2007; Kurowska et al., 2016). While OF1-*En1*^+/−^ mice display the spontaneous neurodegenerative phenotype, *En1*^+/−^ C57Bl/6j (C57-*En1*^+/−^) mice require an additional deletion of *En2* to develop a neurodegenerative phenotype (Sgadò et al., 2006). Since EN2 protein has been shown to protect midbrain dopaminergic neurons from complex I insults and oxidative stress (Alvarez-Fischer et al., 2011; Rekaik et al., 2015b) and can compensate *En1* loss (Hanks et al., 1995; Simon et al., 2001), a hypothesis is that this compensatory effect is more pronounced in C57-*En1*^+/−^ than in OF1-*En1*^+/−^ mice. However, our previous genetic mapping of the susceptibility to dopaminergic neurodegeneration in F2 mice from an OF1-*En1*^+/−^ and C57 intercross showed that the loci linked to dopaminergic neurodegeneration are located outside the *En1* and *En2* loci. We found that the protective effect of the C57 background genome is complex, and maps to 23 partly overlapping and interacting quantitative trait loci (QTL) linked to dopaminergic cell loss in SNpc and axonal pathology (Kurowska et al., 2016).

Here, we aimed to identify processes and pathways associated with susceptibility and resistance to PD-like pathology in *En1*^+/−^ mice with different genetic backgrounds. To achieve this, we analyzed transcriptomic profiles of SNpc neurons from OF1-*En1*^+/−^, C57-*En1*^+/−^ and their respective wild-type (WT) strains at 1 week of age, prior to neurodegeneration. Our results provide evidence that increased expression of genes encoding mitochondrial proteins regulating oxidative phosphorylation protects C57-*En1*^+/−^ mice from dopaminergic neurodegeneration, further supporting energy metabolism as an important player and therapeutic target in PD.

## 2 Materials and methods

### 2.1 Animal husbandry, breeding scheme, and experimental setup

**Figure 1:**
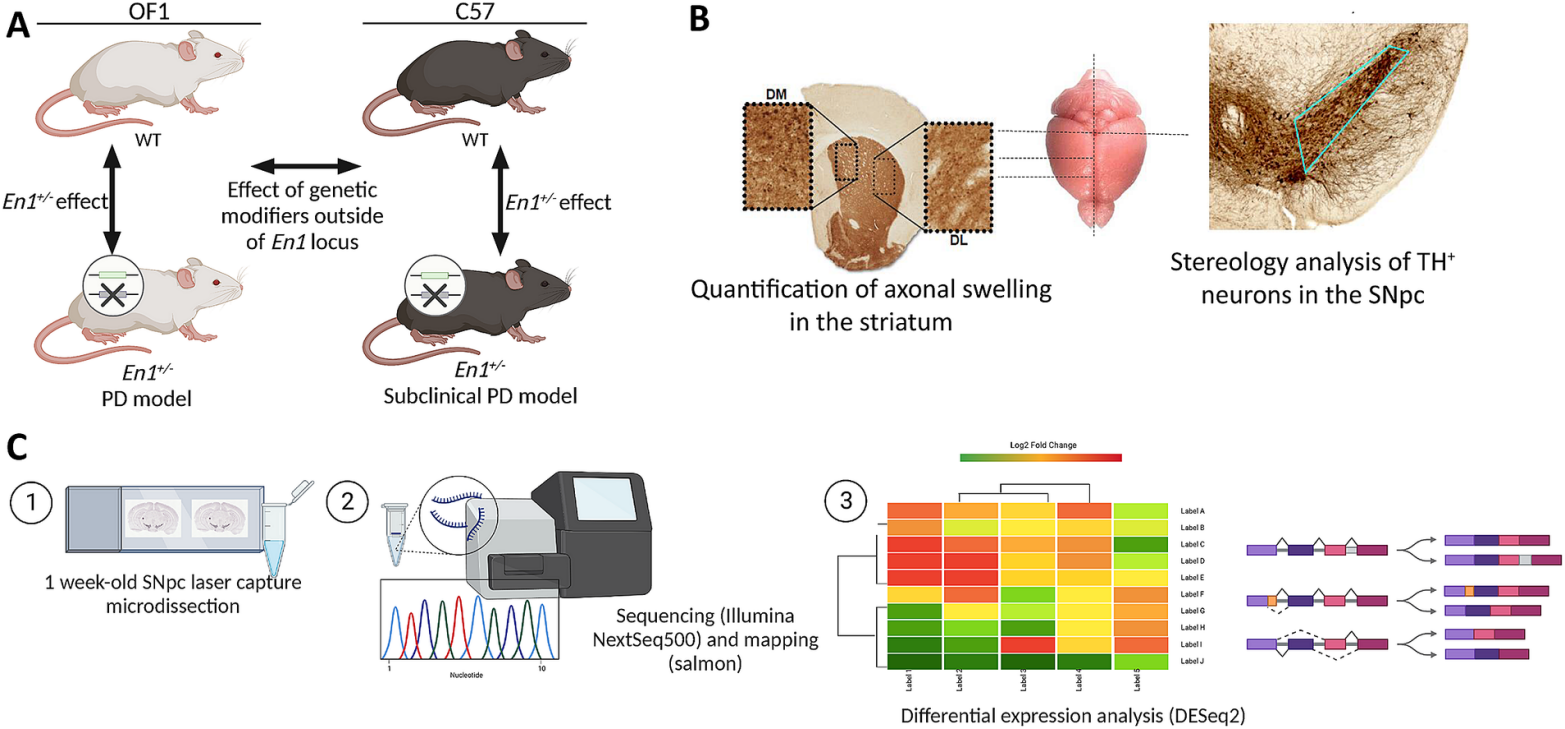
Analysis of genetic background effects on neurodegeneration in the *Engrailed 1* hemizygous (*En1^+/−^*) mouse model for Parkinson’s disease (PD). **(A)** Study design and experimental setup (*n* = 3). **(B)** Sections used for histological analyses of PD-like neuropathology in *En1^+/−^* mice. **(C)** Methodology for bulk RNA-Seq analysis of laser capture microdissected substantia nigra pars compacta (SNpc) from 1 week-old mice. OF1: SwissOF1; C57, C57Bl/6J; TH, tyrosine hydroxylase.

All the experiments were approved by the local ethics committee Malmö/Lund region and specified in permit 4992/2022. Male mice were housed under a 12-h Light: Dark cycle with free access to food and water. Wild-type (WT) Females for the Oncins France 1 (OF1) and C57Bl/6 J (C57) strains were obtained from Charles River Laboratories, France, and mated with *En1*^+/−^ males (C57-*En1*^+/−^ and OF1-*En1*^+/−^). OF1-*En1*^+/−^ were generated as described earlier (Sonnier et al., 2007). C57-*En1*^+/−^ mice were generated by repeated backcrossing (>10 generations) of OF1-*En1*^+/−^ to the C57 background (Van Andel Institute, MI, United States). Mice were sacrificed at 4 different ages: 1, 4, and 16 weeks. Seven mice per genotype and strain were used for histological analysis. As for RNA-Seq, 6 mice from each strain were used, of which 3 were WT and 3 *En1*^+/−^. Exclusively male mice were used in this study. Four groups of male mice were studied to assess both *En1*^+/−^ and genetic background effects (Figure 1A).

### 2.2 Ethics approval

All the experiments were approved by the local ethics committee Malmö/Lund region and specified by permit 4992/2022.

### 2.3 DNA isolation and genotyping

Ear or tail punches were used as the source of genetic material. KAPA® HotStart mouse genotyping kit (KK7351, Roche, CH) was used to extract genomic DNA and perform the PCR reaction following the manufacturer’s instructions. *En1* hemizygosity was determined using primers specific for *lacZ*, inserted in the *En1* locus, producing a null allele (Supplementary Table S1). Amplification conditions were set up as follows: 95°C for 3 min, 32 cycles of 95°C for 30s, 58°C for 30s, 72°C for 30s, followed by a final extension at 72°C for 7 min. To identify the sex of 1-week mice, PCR (94°C for 2 min, 32 cycles of 95°C for 30s, 55°C for 30s, 72°C for 30s, followed by a final extension at 72°C for 7 min) with Y-chromosome specific primers were used (Supplementary Table S1). PCR- products were analyzed on 2% agarose gel with SYBR™ Safe DNA Gel Stain (X10000, #S33102, Thermo Scientific™).

### 2.4 Gene expression analysis with RT-qPCR

RNA from 1 week-old mice ventral midbrain was extracted using TriZol reagent (Invitrogen) according to manufacturer’s instructions. cDNA was generated from 2.5 μg of RNA using the SuperScript III system (Invitrogen) following the manufacturer instructions. A qPCR reaction mix was prepared (5 μL SSoAdvance Universal SYBR®Green Supermix (Biorad) 0,5 μL primer Forward, 0,5 μL primer Reverse and 4 μL cDNA), followed by. PCR (95°C 40s, 45 cycles of 95°C 15 s, 60°C 30s) and a melt curve between 65 and 95°C with an increment of 0.5°C for 5 s (CFX96, BioRad).

Primers were designed using Primer-BLAST (NCBI) aiming to get 60°C melting temperature and product length of 100–200 bp. Primers used were *En1* (forward 5′-GTG GTC AAG ACT GAC TCA CGC-3′, reverse 5’-GCT TGT CTT CCT TCT CGT TCT T-3′), *R18s* (forward 5’-GCA CAG TGT TTG TAG AGC CTG-3′, reverse 5’-GCC CTG GAA CTT ATT GAT CGG G-3′) and *HPRT* (forward 5’-AGC AGG TGT TCT AGT CCT GTG G-3′, reverse 5’-ACG CAG CAA CTG ACA TTT CTA A-3′).

### 2.5 Immunohistochemistry, axonal swelling quantification, and stereology

Histological evaluation was performed for 4- and 16-week-old mice, since axonal pathology is evident at 4 weeks, neurodegeneration in SNpc starts at 6 weeks and is established at 16 weeks (Sonnier et al., 2007; Nordström et al., 2015; Rekaik et al., 2015a). Mice were euthanized by intraperitoneal injection of 0.2 mL of sodium pentobarbital (60 mg/mL), perfused with ice-cold saline (0.9% NaCl) for 5 min, and fixated by perfusion with 4% paraformaldehyde (PFA, pH7.4) for 5 min. Brains were then removed and postfixed in 4% PFA overnight (O.N.). Once fixated, brains were transferred into a 30% sucrose, with 0.01% sodium azide solution in phosphate buffer (PBS) until fully embedded.

For immunohistochemistry (IHC) brains were sectioned coronally on a freezing sled microtome (Leica SM2010R). Six series of consecutive coronal 40 μm thick sections were collected for both striatum and SNpc (Figure 1B). Sections were stored at 4°C on Walter’s antifreeze (30% (v/v) ethylene glycol, 30% (v/v) glycerol,0.01% (v/v) sodium azide, and 0.5 M phosphate buffer) until tyrosine hydroxylase (TH) staining. Free-floating sections were washed 3 times with PBS and quenched for 30 min with 3%H_2_O_2_/10% MeOH in PBS. After washing again with PBS, sections were permeabilized by incubating for 10 min in 0.3% Triton X-100 in PBS (PBS-T) and blocked with 5% normal goat serum for 2 h. Next, sections were stained O.N., at 4°C, with TH primary antibody (1:4000, AB152, Millipore). The next day, sections were washed with PBS-T and incubated with a biotinylated secondary antibody (1:200, BA9200, Vector Laboratories, United Kingdom) for 1 h at room temperature. This was followed by washes with PBS-T and PBS, and a 30 min incubation with Avidin-Biotin complex reagent (ABC Elite, Vector Laboratories, United Kingdom). Finally, immunostaining was revealed by incubation with diaminobenzidine (DAB) as the chromogen. Mounted sections were then dehydrated on increasing concentrations of ethanol, cleared with xylene, and slipcovered using DPX mounting media.

Axonal swelling quantification was done following previous analysis done in the same animal model (Nordström et al., 2015). Three consecutive sections from each animal were used. Pictures were taken at ×20 magnification using an Olympus BX53 microscope (Olympus, Japan). Two pictures per section were taken representing the dorsal part of the caudate putamen. Analysis was done using ImageJ (1.52a, Java 1.8.0_112 64-bits, NIH, United States). Axonal swelling was counted and classified according to their size: small (0.1–1.5 μm), medium (1.51–2.99 μm), and large (>3 μm). Averages were determined based on the total pictures for each animal.

Stereology analysis was done following the optical fractionator principle (West and Gundersen, 1990) to estimate the total number of TH^+^ dopaminergic neurons in the SNpc. Analysis was done on every third section of the midbrain of each animal, resulting in 8–10 sections per animal. For imaging and quantification, a Leica MPS52 microscope and the Stereo Investigator® software (MBF Bioscience) were used. The parameters include a 5X/0.11 lens for delimiting the region of interest (ROI), 100X/1.3 lens for counting, a counting frame size of 55 × 55 μm, a sampling area of 130 × 130 μm, a section thickness of 30.1 ± 0.9 μm and a Gundersen coefficient error of ≤0.07.

### 2.6 Laser capture microdissection, RNA extraction from nigral tissue, RNA sequencing, and read counting

Transcriptional analyses were performed on SNpc from 1-week old mice to reflect gene expression prior to neurodegeneration. The time point was selected since the neurodegenerative process precedes quantifiable cell loss in SNpc in OF1-*En1*^+/−^ mice; nigrostriatal axonal pathology appears and progressively increases already from postnatal day 8 and TH and dopamine is significantly reduced in the striatum at 4 weeks (Nordström et al., 2015). One-week-old mice were sacrificed by decapitation; brain dissections were performed on ice in under 2 min to preserve RNA integrity. Once removed from the skulls, the brains were embedded in Optimal Cutting Temperature Compound (Tissue-Tek® O.C.T. Compound, Sakura® Finetek), snap-frozen in liquid nitrogen, and stored at −80°C until used.

Before the microdissection, Polyethylene naphtholate (PEN)-membrane slides (1 mm, 0.17 mm; 415190-9041-000, Carl Zeiss Microimaging, Inc.) and staining jars were heated at 180°C for 4 h followed by UV light irradiation to completely inactivate RNases and destroy contaminating nucleic acids. Frozen tissue blocks were transferred to a cryostat (CM3050 S, Leica Microsystems) that was pre-set to −17°C and allowed to equilibrate for at least 1 h before sectioning. A total of six 14 μm-thick sections containing early substantia nigra (approximately equivalent to Bregma −2.92 to −3.16 in adult mice) were collected from each experimental animal, accommodating three per slide. Slides were stored at −80°C until staining and Laser Capture Capture Microdissection (LCM), which were performed on the same day. A short staining procedure was conducted, and ice-cold solutions were used to preserve the RNA integrity. Briefly, sections were fixed in 70% EtOH for 2 min, followed by staining with 1% w/v cresyl violet acetate prepared in 50% EtOH. After the removal of excess stain, the slides were dipped in 70 and 100% EtOH and air-dried before capturing the target region using the PALM Robot Microbeam Laser Microdissection System (Zeiss) at the Division of Oncology and Pathology (Department of Clinical Sciences, Lund University).

AdhesiveCap clear tubes (415190-9211-000, Zeiss) were used for collecting the nigral tissue from each animal separately: approximately 0.3 mm^2^ of tissue was harvested from each animal and catapulted onto the adhesive cap. Immediately after LCM, tissue lysis was performed using 200 μL RLT buffer with β-mercaptoethanol from the RNeasy Micro kit (#74004, QIAGEN,) in a ventilated hood for 30 min followed by centrifugation at 9000rcf for 5 min. Total RNA purification was performed following the RNeasy Micro kit. The quality of the RNA was assessed on an Agilent 2,100 Bioanalyzer RNA following the manufacturer’s instructions (G2946-90005, Agilent Technologies, Inc.). Samples were then stored at −80°C until cDNA library preparation and sequencing.

Library construction and sequencing were performed at the MultiPark Next Generation Sequencing facility (Department of Experimental Medical Science, Lund University). Due to the low initial RNA concentrations, the SMART-Seq^®^ v4 Ultra^®^ Low Input RNA Kit (Takara Bio, Inc.) was used for cDNA library preparation; the output was then processed with the Nextera® XT DNA Library Preparation Kit (Illumina, Inc.) for paired-end (2 × 75 bp) sequencing on an Illumina NextSeq500 instrument. Multiplex libraries were sequenced at an average depth of 20 million reads per sample.

After demultiplexing, the quality of the Illumina reads stored in FASTQfiles was assessed by means of FastQC (v0.11.4). Transcript quantification was performed using Salmon (v0.8.2) and the transcriptome-based quasi-mapping model (Patro et al., 2017). The following flags were used for quasi-mapping: *--gcBias* and *--validateMappings*. A Bash script was used to run Salmon on all samples in serial. The transcript index was derived from the GENCODE mouse release M28 (GRCm39) containing nucleotide sequences of all transcripts on the reference chromosomes.

After transcript quantification, the data were imported into the R statistical computing environment (v4.1.2) and summarized using tximport (v1.22) (Figure 1C).

### 2.7 Identification of differentially expressed genes

Differential gene expression analysis between (i.e., OF1 vs. C57) and within (i.e., En1^+/−^ vs. WT) strains was done using DESeq2 (v1.32) following the authors’ suggested workflow (Love et al., 2014). Transcripts that did not reach a threshold of 10 normalized read counts after averaging across biological replicates were eliminated and considered as not expressed to reduce noise and increase power. The model for differential gene expression was parameterized to evaluate strain and genotype effect, and its interactions (~Genoype + Strain + Genotype:Strain). Contrasts for each of the 4 comparisons (Figure 1A) were analyzed. Differential expression was considered significant at a Benjamini-Hochberg-adjusted *p* < 0.05.

### 2.8 Functional enrichment analysis of DEGs

The clusterProfiler (v4.0.5) package (Yu et al., 2012) was used to explore overrepresented biological pathways using the Gene Ontology (GO), Kyoto Encyclopaedia of Genes and Genomes (KEGG) and Wikipathways. GO categories with a Benjamini-Hochberg-corrected *p* ≤ 0.05 are reported. Reactome pathway-based analysis was done using the ReactomePA package (Yu and He, 2016). The database was accessed in January 2023 and *p*adj < 0.05 was used for significance threshold.

### 2.9 Experimental design and statistical analyses

Statistical tests for the striatal axonal swelling quantification and stereological counts of TH^+^ cells in SNpc of 4- and 16-week-old mice were performed using GraphPad Prism Software (version 7, GraphPad, La Jolla, CA). The Shapiro–Wilk test was performed in R (v4.1.2) to test for normality. Differences between groups were analyzed using two-way ANOVA with Tukey’s multiple comparisons test; statistical significance was set at *p* < 0.05 and values are expressed as mean ± standard deviation (SD) for the histological analyses.

Pearson’s correlation coefficient (*r*) was used to assess the linear relationship between striatal load of axonal swellings and the number of TH^+^ neurons in the SNpc. The significance level was set at *p* < 0.05, *r* and *r*^2^ are reported. To further explore the relationship, linear regression analyses were conducted. The slope of the regression line, representing the change in load of axonal swellings per additional TH^+^ neuron, was calculated to quantify the strength of the association. The significance of the regression coefficients was assessed to determine whether the relationship remained statistically significant.

### 2.10 Data availability

The datasets generated for this study can be found in the GEO repository under the accession number GSE236461. Further data can be made available upon request. All further data supporting the conclusions of this article are included within the article and in additional files provided.

## 3 Results

### 3.1 Both OF1-*En1*^+/−^ and C57-*En1*^+/−^ mice display early nigrostriatal pathology

**Figure 2:**
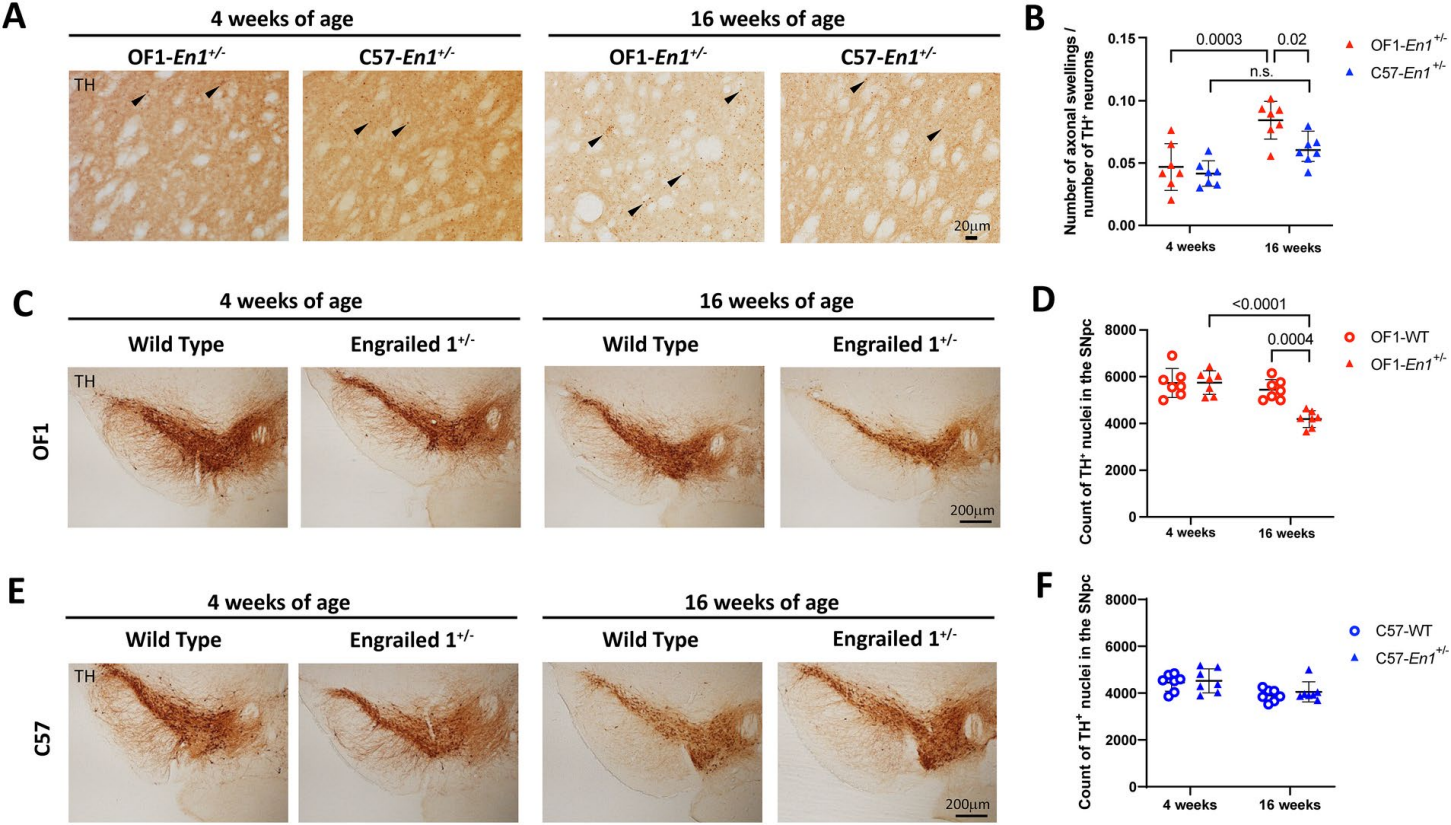
Engrailed 1 hemizygosity (*En1^+/−^*) induces progressive nigrostriatal dopaminergic neurodegeneration in OF1 but not C57 mice. **(A)** Representative images of axonal swellings in the striatum of OF1-*En1^+/−^* and C57-*En1^+/−^* mice at 4 and 16 weeks-of-age. **(B)** In OF1-*En1^+/−^*mice, there is a significant increase in the number of swellings per remaining dopaminergic neuron in the substantia nigra pars compacta (SNpc) between 4 and 16 weeks, while axonal swellings do not increase in C57-*En1^+/−^* mice. Data presented as mean ± SD (Two-way ANOVA with Tukey’s multiple comparison test, *n* = 7). **(C)** Representative images of SNpc from OF1 wild type (WT) and OF1-*En1^+/−^* mice at 4 and 16 weeks-of-age. **(D)** The number of dopaminergic neurons in the SNpc is significantly lower in 16 week-old OF1-*En1^+/−^* mice compared to both 16 week-old OF1 WT and 4 week-old OF1-*En1^+/−^* mice. **(E)** Representative images of SNpc from C57 WT and C57-*En1^+/−^* mice at 4 and 16 weeks-of-age. **(F)** C57-*En1^+/−^* mice did not display significant loss of dopaminergic neurons in the SNpc at 16 weeks. TH, tyrosine hydroxylase; OF1, SwissOF1; C57, C57Bl/6J.

Previous work has shown signs of nigrostriatal degeneration in OF1-*En1*^+/−^ mice in the form of spheroidal dystrophic terminals (axonal swellings) in 4 and 16 weeks-old mice, with no significant differences in 8 days-old pups (Sgadò et al., 2006; Sonnier et al., 2007; Nordström et al., 2015). We found similar levels of striatal axonal swellings per number of TH+ neurons in SNpc at 4 weeks in OF1-*En1*^+/−^ and C57-*En1*^+/−^ mice (0.047 ± 0.019 vs. 0,042 ± 0.010; n = 7) (Figures 2A,B). While axonal swellings had significantly increased at 16 weeks compared to 4 weeks in OF1-*En1*^+/−^ mice (0.084 ± 0.015 vs. 0.047 ± 0.019, p = 0.0003), they did not increase in C57-*En1*^+/−^ mice (0.061 ± 0.012 vs. 0.042 ± 0.010, p = 0.09). Furthermore, OF1-*En1*^+/−^ had significantly more axonal swellings compared to C57-*En1*^+/−^ mice at 16 weeks (0.084 ± 0.015 vs. 0.061 ± 0.012, *p* = 0.02). Two-way ANOVA indicated effects of age (*F* (1, 24) = 27.2, *p* < 0.0001) and strain (*F* (1, 24) = 7.28, *p* = 0.01), but no interaction between the two factors.

Early nigrostriatal pathology is thus manifested in both strains, but while the axonal pathology increases over time in OF1-*En1*^+/−^ mice, it does not progress in C57-*En1*^+/−^ mice. This suggests that genetically determined compensatory mechanisms can counteract a progressive PD-like phenotype.

### 3.2 C57 genetic background protects against dopaminergic neuron loss induced by *En1* hemizygosity

We have previously reported that the loss of dopaminergic neurons due to *En1* hemizygosity is genetically determined by multiple QTLs (Kurowska et al., 2016). Stereological counts of TH+ neurons in the SNpc showed no significant difference at 4 weeks (mean number OF1-En1^+/−^ 4w 5,746 ± 463.5 vs. OF1 WT 4w 5,732 ± 572.1, *p* = 1.0) but 23% fewer dopaminergic neurons at 16 weeks in OF1-*En1*^+/−^ compared to OF1 WT mice (mean number 4187 ± 355.8 vs. 5,442 ± 432.9, *p* = 0.0004) (Figures 2C,D). Two-way ANOVA showed significant effects of age (*F* (1, 24) = 25.23, *p* < 0.0001), *En1*^+/−^ genotype (*F* (1, 24) = 11.36, *p* = 0.0004) and interaction between the two (*F* (1, 24) = 11.89, *p* = 0.0021) on nigral dopaminergic cell counts in OF1 mice. In contrast, C57-*En1*^+/−^ mice showed no loss of dopaminergic neurons in the SNpc when compared to C57 WT at 4 weeks (4,525 ± 514.4 vs. 4,451 ± 369.0, *p* = 1.0) or 16 weeks (4,053 ± 428.5 vs. 3,902 ± 263.9, *p* = 0.9) (Figures 2E,F). Two-way ANOVA detected a significant effect of age (*F* (1,24) = 11.13, *p* = 0.0028), but not for *En1*^+/−^ genotype or any interaction between the two for C57 mice.

We have previously shown a strong negative correlation between TH+ cell counts in SNpc and the load of axonal swellings in the striatum (axonal swellings relative to TH+ cell count) in an intercross between OF1-*En1*^+/−^ and C57 mice (Kurowska et al., 2016). We confirm a negative correlation at 16 weeks for OF1-*En1*^+/−^ (*r*^2^ = 0.44, *p* = 0.005; Supplementary Figure S1A). The correlation is, however, absent in C57-*En1*^+/−^ (*r*^2^ = 0.20, *p* = 0,051; Supplementary Figure S1B). Further, regression analyses defined the slope as significantly non-zero for OF1-En1^+/−^ (*p* = 0.0097) but not for C57-En1^+/−^ (*p* = 0.10).

Histology thus confirms progressive dopaminergic neurodegeneration in OF1-*En1*^+/−^ mice and that the genetic background in C57-*En1*^+/−^ mice confers resistance to this PD-like phenotype. Of note, the estimated number of dopaminergic neurons in the SNpc was lower in C57-WT mice compared to OF1-WT at both 4 and 16 weeks, consistent with our previous findings (Kurowska et al., 2016).

### 3.3 Transcriptomic analysis of laser-captured micro-dissected SNpc

To assess factors driving or counteracting the neurodegenerative process in its early stages, we performed RNA-sequencing of laser-captured micro-dissected (LCM) SNpc from 1 week-old OF1-WT, OF1-*En1*^+/−^, C57 WT and C57-*En1*^+/−^ mice (*n* = 3 per group). We obtained high-quality RNA (RIN > 8.0). All samples showed high Phred quality scores (>30) after sequencing, as assessed by FastQC (v0.11.8) software with most of the reads being 75 bp long.

Principal components analysis (PCA) showed that the main effect on transcriptome profiles in the dataset was due to genetic background (Supplementary Figure S2A). Some of the DEGs appeared in more than one comparison (intra-strain or between-strain; Supplementary Figure S2B).

Many of the transcripts routinely used for the identification of intermediate progenitors or immature neurons had very low expression levels at this stage (*Pax6, Dcx, NeuroD1, Tbr1*) with some of them being undetectable (*Tbr2, Ngn2*). Expression of transcripts associated with mature dopaminergic neurons was consistent across all the groups (*Aldh1a1, Lmx1B, Th, Slc6a3, Girk2*), except for *Foxa2*, showing a lower expression in C57-*En1*^+/−^ mice compared to both C57-WT and OF1-*En1^+/−^* mice. A decreased expression of *En1* was seen in both OF1-*En1*^+/−^ and C57-*En1*^+/−^ mice compared to their respective WT controls, but low counts bearing high dispersion resulted in no significant differences (Supplementary Figure S2C). *En2* expression was not detected, discarding any compensatory effects. This is in accordance with quantitative PCR data, showing a low and varying expression of *En1* and no strain differences in *En2* expression (Supplementary Figure S2D).

### 3.4 Distinct transcriptomic profiles are induced by *En1* hemizygosity in OF1 and C57 mice

**Figure 3:**
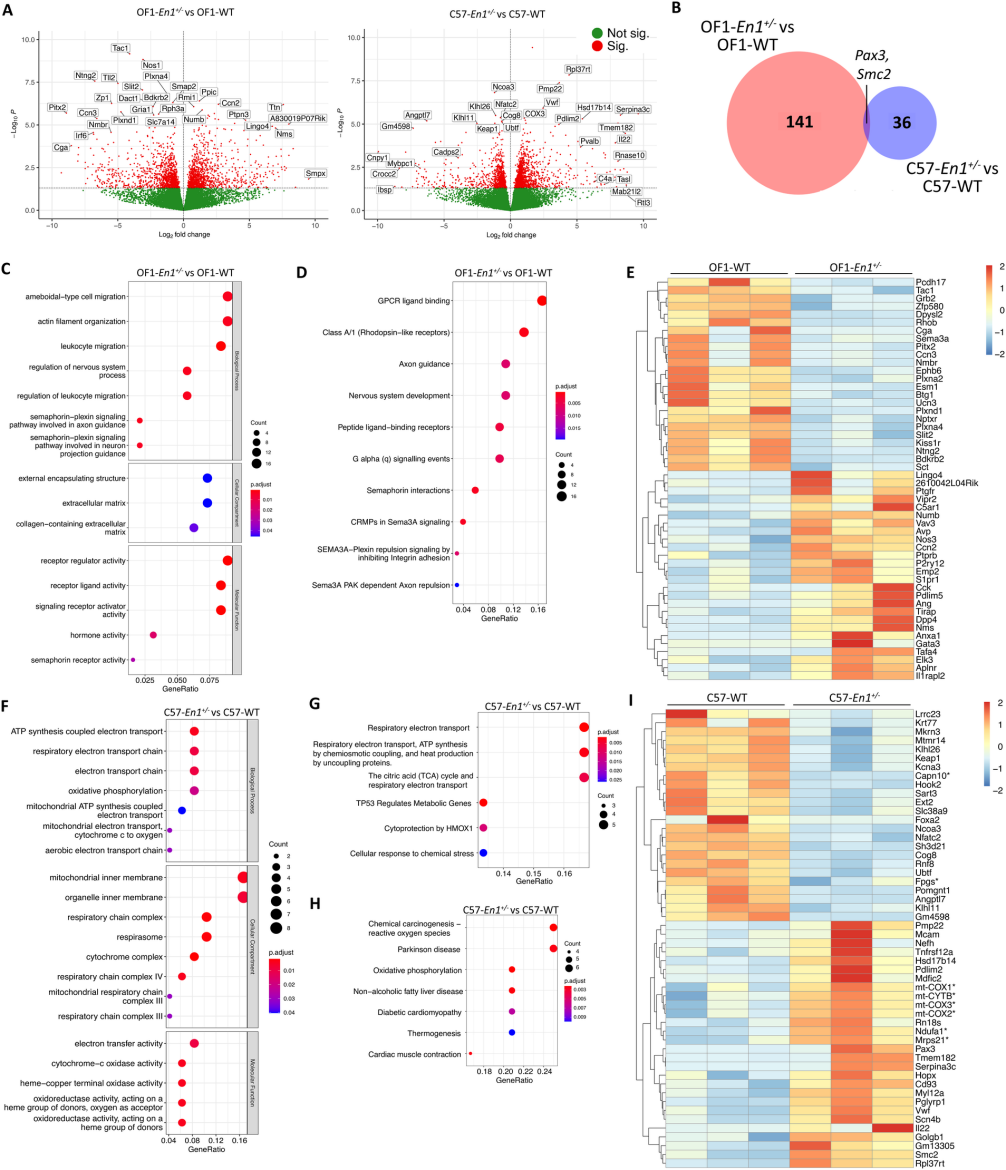
Engrailed-1 hemizygosity (*En1^+/−^*) effects on nigral transcriptomic profiles in OF1 and C57 mice. **(A)** Volcano plots showing all expressed genes in comparisons OF1-*En1^+/−^*vs. OF1 wild-type (WT) mice and C57- *En1^+/−^* vs. C57 WT mice. Vertical lines indicate fold change (|FC|) of 1.5. **(B)** Venn diagram illustrating differentially expressed genes (DEGs) in OF1*-En1^+/−^* vs. OF1 WT and in C57*-En1^+/−^* vs. C57 WT, overlapping only by 2 DEGs. **(C)** Gene Ontology (GO) enrichment analysis of DEGs in OF1-*En1^+/−^* vs. OF1 WT mice. **(D)** Reactome pathway analysis for DEGs in OF1-*En1^+/−^* vs. OF1 WT mice. **(E)** Heatmap of enriched pathway genes in OF1-*En1^+/−^* vs. OF1 WT mice. OF1-*En1^+/−^* mice show a higher expression of genes associated with neuron development and axonal guidance. **(F)** GO enrichment analysis of DEGs in C57-*En1^+/−^* vs. C57 WT mice. **(G)** Reactome pathway analysis for DEGs in C57-*En1^+/−^* vs. C57 WT mice. **(H)** KEGG pathway enrichment analysis for DEGs in C57-*En1^+/−^* vs. C57 WT mice, including Parkinson’s disease. **(I)** Heatmap of enriched pathway genes in C57-*En1^+/−^* vs. C57 WT mice. C57- *En1^+/−^* mice show a higher expression of genes encoding mitochondrial proteins (marked with *). For gene symbols, *mt* stands for genes encoded by the mitochondrial genome. Red: up-regulated, blue: down-regulated. GO terms were separated according to their Ontology category (Biological Process, Cellular Compartment and Molecular Function). OF1, SwissOF1; C57, C57Bl/6J.

Firstly, we aimed to study the effects of *En1* hemizygosity on transcriptional profiles in OF1 and C57 mice. *En1* hemizygosity induced more transcriptional changes in OF1 than in C57 mice; 198 DEGs for OF1-*En1*^+/−^ vs. OF1 WT and 52 DEGS for C57-*En1*^+/−^ vs. C57 WT (FDR < 0.05, Figures 3A,B). Among the DEGs, 141 were exclusive to OF1 and 36 were exclusive to C57. Only two genes (*Pax3* and *Smc2*) were differentially expressed in both OF1-*En1*^+/−^ and C57-*En1*^+/−^ compared to WT mice (Figure 3B; Supplementary Figure S2B).

We performed enrichment analyses with GO, KEGG and Wikipathways on the DEGs identified in OF1-*En1*^+/−^ versus OF1 WT to get insight into biological pathways induced by *En1*^+/−^, along with their associated molecular functions and cellular localization (Additional file 2). Among the most enriched biological processes (GO) were actin filament organization (*q* value 0.002), semaphorin-plexin signaling involved in axon guidance (*q* value 0.002) and leukocyte migration (*q* value 0.004), all of which play key roles in brain development by modulating axonal guidance and cell migration (Limoni and Niquille, 2021) (Figure 3C). The KEGG database did not identify enriched pathways, but Reactome pathway-based analysis identified enriched pathways linked to axon guidance, nervous system development and semaphoring-dependent signaling, further reinforcing the GO enrichment results (Figure 3D). Expression of genes involved in axonal cone growth and guidance, as well as nervous system development, were lower in OF1-*En1*^+/−^ compared to OF1 WT mice. DEGs present in several of the enriched pathways include noteworthy candidates involved in neuronal development and survival, including *Cck*, *Anxa1*, *Nos1*, *Nos3*, *Plxna2*, *Plxna4*, *Plxnd1,* and *Sema3a* (Figure 3E).

Despite the low number of DEGs in C57-*En1*^+/−^ vs. C57 WT mice, GO enrichment analysis identified biological processes linked to ATP generation in mitochondria. For the cellular compartment category, the enrichment was related to the mitochondrial inner membrane and specifically the respiratory chain complex (Figure 3F). Reactome pathway analysis also identified pathways associated with energy metabolism in mitochondria, including respiratory electron transport, ATP production and tricarboxylic acid cycle (Figure 3G). Interestingly, pathways related to reactive oxygen species (ROS) production, oxidative phosphorylation (OxPhos), and neurodegenerative diseases were enriched in C57-*En1*^+/−^ vs. C57 WT mice according to KEGG (Figure 3H). Of note, genes related to OxPhos and ATP generation including *Ndufa1* (part of complex I), *mt-Cytb* (part of complex III), *mt-Co1, mt-Co2, mt-Co3* (constituents of complex IV) along with constituents of mitochondrial ribosomes (*Mrps21*) all had a higher expression in C57-*En1*^+/−^ compared to C57 WT mice (Figure 3I).

### 3.5 C57 mice display uniquely regulated genes related to the electron transport chain in mitochondria

**Figure 4:**
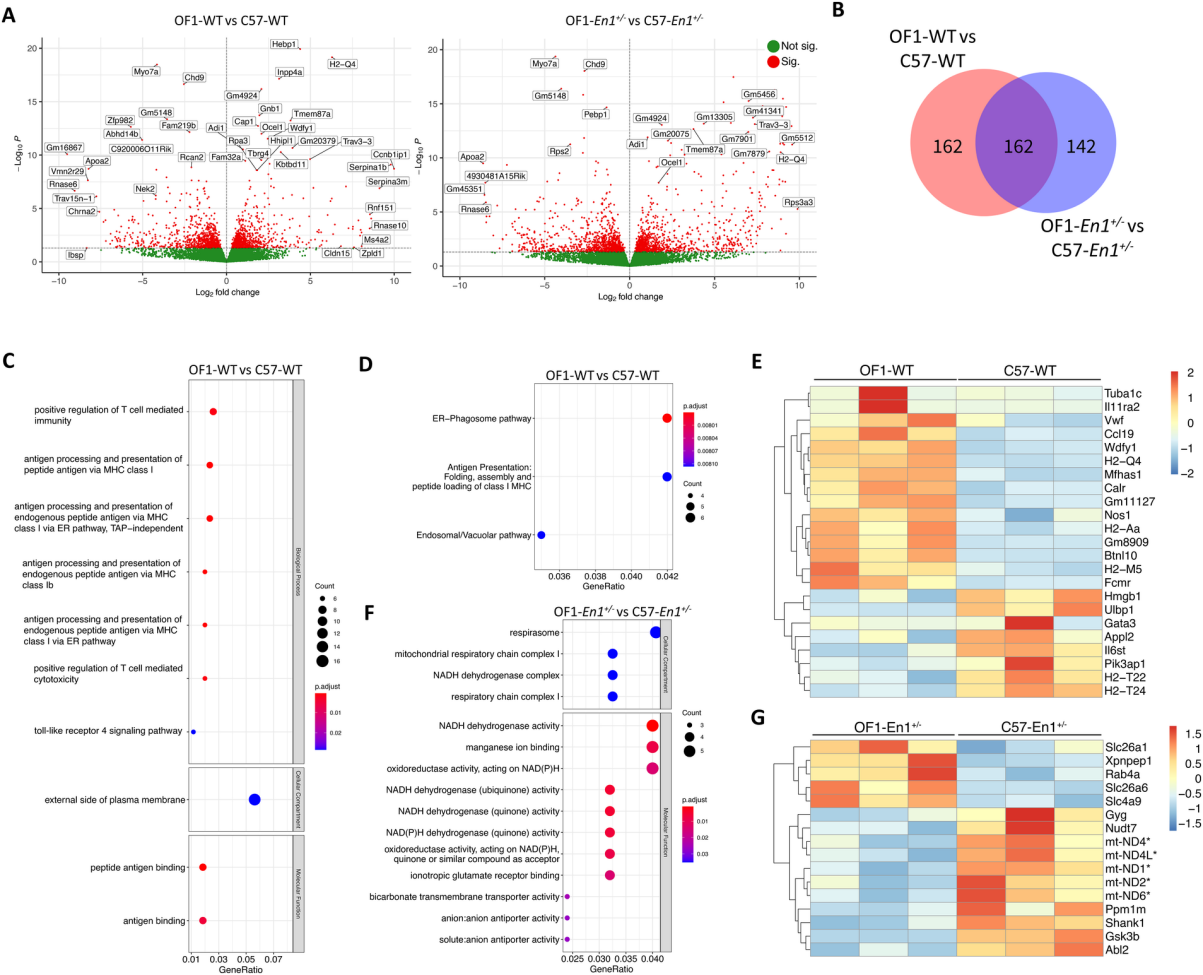
Transcriptomic differences between OF1 wild-type (WT) and C57 WT mice as well as OF1 *Engrailed-1* hemizygous (*En1^+/−^*) and C57-*En1^+/−^* mice. **(A)** Volcano plots showing all expressed genes in OF1 WT vs. C57 WT mice and in OF1-*En1^+/−^* vs. C57*-En1^+/−^* mice. Vertical lines indicate fold change (FC) of 1.5. **(B)** Venn diagram differentially expressed genes (DEGs) in OF1 WT vs. C57 WT and in OF1*-En1^+/−^* vs. C57*-En1^+/−^* mice. **(C)** Gene Ontology (GO) enrichment analysis of DEGs in OF1 WT vs. C57 WT mice. **(D)** Reactome pathway analysis for DEGs in OF1 WT vs. C57 WT mice shows pathways associated with vesicular trafficking and antigen presentation. **(E)** Heatmap of enriched pathway genes in OF1 WT vs. C57 WT mice. OF1 WT mice show a higher expression of genes associated with antigen presentation and vesicle trafficking. **(F)** GO enrichment analysis of the 142 DEGs exclusive to OF1-*En1^+/−^* vs. C57-*En1^+/−^* mice shows enrichment in GO terms associated to energy metabolism in mitochondria. **(G)** Heatmap of enriched pathway genes in OF1-*En1^+/−^* vs. C57-*En1^+/−^* mice. C57*-En1^+/−^* mice consistently show higher expression of genes associated with mitochondrial respiration and oxidative phosphorylation (marked with*). For gene symbols, *mt* stands for genes encoded by the mitochondrial genome. Red: up-regulated, blue: down-regulated. GO terms were separated according to their Ontology category (Biological Process, Cellular Compartment and Molecular Function). OF1, SwissOF1; C57, C57Bl/6J.

We found 366 DEGs between OF1 WT and C57 WT and 335 DEGs between OF1-*En1*^+/−^ and C57-*En1*^+/−^ mice, with 162 DEGs independent of *En1*-hemizygozity (Figures 4A,B).

To identify gene expression changes and enriched pathways associated with strain-dependent susceptibility to PD-like pathology, we compared transcriptional profiles in SNpc of OF1 and C57 mouse strains at 1 week. Between the WT strains, there were 365 DEGs, 210 with higher and 156 with lower expression in OF1 WT compared to C57 WT mice. Between *En1*^+/−^ mice, there were 334 DEGs, 184 with higher and 151 with lower expression in OF1-*En1*^+/−^ compared to C57-*En1*^+/−^ mice (Figure 4B; Supplementary Figure S2). Among the 701 transcripts differing between OF1 WT vs. C57 WT and/or OF1-*En1*^+/−^ vs. C57-*En1*^+/−^, 162 DEGs were regulated by the strain background, independent of *En1*^+/−^ (Figure 4B).

The 366 transcripts with differential expression in OF1 WT vs. C57 WT were enriched in GO-terms including antigen processing and presentation via major histocompatibility complex (MHC) class I, positive regulation of T cell-mediated cytotoxicity, and toll-like receptor 4 signaling pathway (Figure 4C). Reactome pathway analysis showed that ER-phagosome and antigen presentation pathways were enriched, thus further supporting the GO enrichment results (Figure 4D). Among the genes present in the enriched pathways were genes encoding proteins involved in antigen presentation, including *H2-Qa*, *H2-Aa*, *Gm11127*, *H2-T22*, and *H2-T24* (Figure 4E).

No enrichment by GO, KEGG, or Reactome pathway enrichment analysis was observed for the 335 DEGs between OF1-*En1*^+/−^ and C57-*En1*^+/−^ mice. However, the 167 DEGs that were exclusive to *En1*^+/−^ and not differing between the WT strains, were significantly enriched in molecular functions related to NADH dehydrogenase activity and ion transporter activity. No biological processes were significantly enriched; however, it is worth noting that enrichment for cellular compartment ontology showed again localization to the mitochondria, particularly in the respirasome (Figure 4F), like DEGs found between C57-*En1^+/−^* and C57 WT mice. Among these, the expression of OxPhos-related genes was higher C57-*En1*^+/−^ mice compared to OF1-*En1*^+/−^ mice. GO terms like respirasome, mitochondrial respiratory chain complex I and NADH dehydrogenase activity included genes encoding constituents of complex I (*mt-Nd1, mt-Nd2, mt-Nd4, mt-Nd4L, mt-Nd6*) and complex V ATP synthase (*mt-Atp8*). This suggests that C57-*En1*^+/−^, but not OF1-*En1*^+/−^, mice compensate for impairments caused by *En1*^+/−^ at a transcriptional level to increase ATP production.

**Figure 5:**
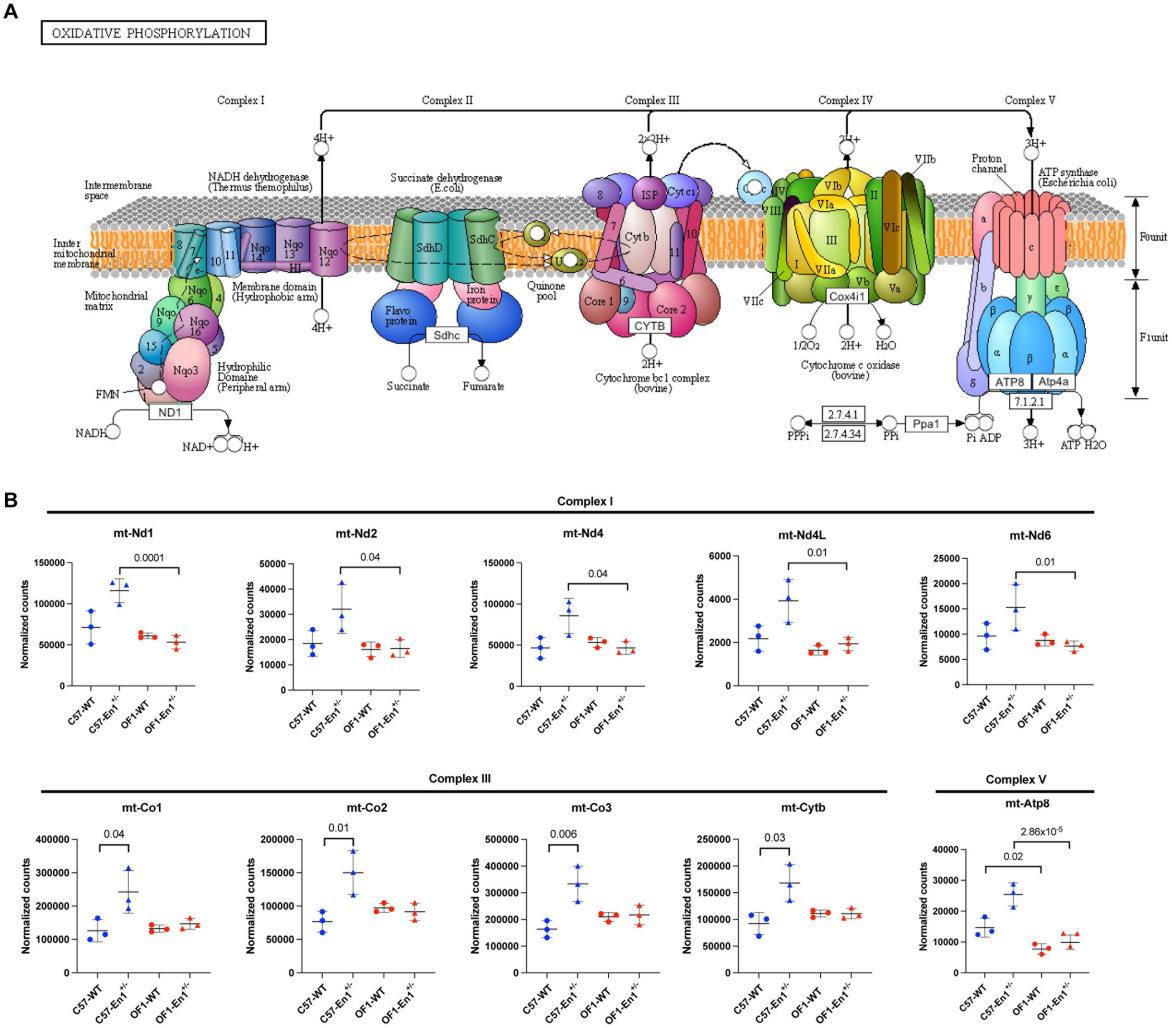
C57 hemizygous (*En1^+/−^*) mice have higher expression of genes encoding components of the oxidative phosphorylation (OxPhos) pathway compared to OF1 *En1^+/−^* and C57 wild-type (WT) mice. **(A)** KEGGs representative diagram of protein complexes in the OxPhos pathway located in the mitochondrial inner membrane. **(B)** Normalized counts of regulated transcripts mapped to the OxPhos-pathway. C57-*En1^+/−^* mice have higher expression of genes coding for complex I and V subunits compared to OF1-*En1^+/−^* mice. C57- *En1^+/−^* mice also have higher expression of genes coding for components of complex III compared to C57 WT mice and C57 WT mice have higher expression of *mtAtp8* (in complex V) compared to OF1 WT mice. OF1, SwissOF1; C57, C57Bl/6J. (Figure 5A) modified from KEGG: Kyoto Encyclopedia of Genes and Genomes, map00190.

Given the enrichment of OxPhos-related genes among DEGs and the importance of energy metabolism and mitochondrial functionality in neurodegenerative diseases, we analyzed normalized counts from RNAseq for OxPhos-related genes in all groups of mice. According to these, there was a significantly higher expression of genes encoding complex I and complex V proteins in C57-*En1*^+/−^ compared to OF1-*En1*^+/−^ mice, a higher expression of genes encoding complex III proteins in C57-*En1*^+/−^ compared to C57 WT mice and higher expression of *mt-Atp8* encoding complex V protein in C57 WT compared to OF1 WT mice (Figure 5).

## 4 Discussion

Characterization of the susceptibility to multifactorial diseases like PD is challenging, partly due to complexity with multiple genetic and environmental factors and interactions between them (Nalls et al., 2019; Bloem et al., 2021). In addition, identification of the cellular and physiological mechanisms that define and mediate this susceptibility is further complicated by the fact that the disease is often diagnosed late in life when the degenerative process is already established. Animal models that recapitulate the underlying and early processes of susceptibility (or resistance) to neurodegeneration are therefore of great importance. Here, we report on a transcriptional profile in young mice that show resistance to progressive PD-like pathology, characterized by an increased expression of genes encoding mitochondrial proteins in the SNpc.

The *En1^+/−^* mouse model has been shown to reproduce many of the characteristic traits of PD, including genetically determined susceptibility to PD-like pathology in different background strains. Previous works have shown that OF1*-En1^+/−^* mice exhibit loss of nigrostriatal dopaminergic neurons (Sonnier et al., 2007; Nordström et al., 2015) while C57*-En1^+/−^* mice lack this PD-like phenotype and require homozygous deletion of *En2* (*En2*^+/−^) to develop a similar neurodegeneration (Sgadò et al., 2006). Furthermore, polymorphisms in the *EN1* gene have been associated with idiopathic PD in a small cohort study (Haubenberger et al., 2011). Our previous genetic linkage analysis in a C57⨯OF1-*En1*^+/−^ F2 intercross identified multiple interacting QTLs linked to PD-like phenotypes induced by *En1* hemizygosity (Kurowska et al., 2016). The complex genetic regulation of the traits makes further QTL mapping difficult and calls for complementary strategies. We therefore decided to use transcriptomic analyses to identify factors underlying the susceptibility to *En1^+/−^*-induced PD-like pathology. To our knowledge, this is the first work to perform a susceptibility study on the SNpc of *En1*^+/−^ mice at a transcriptomic level.

Nigrostriatal axonal pathology is an early characteristic of PD, before the evident loss of neuronal bodies in the SNpc (Burke and O’Malley, 2013; Kordower et al., 2013). Our results show that both OF1-*En1*^+/−^ and C57-*En1*^+/−^ mice exhibit such early signs of neurodegeneration in the form of nigrostriatal axonal swellings at 4 weeks of age, but that progressive axonal pathology and loss of dopaminergic neuron cell bodies in SNpc are only seen in OF1-*En1*^+/−^ mice. Based on the presence of early signs of dopaminergic neurodegeneration but resistance to nigral dopaminergic nerve cell loss, we propose C57-*En1*^+/−^ mice as a suitable model to map and characterize genetic factors mediating resistance to PD-like neurodegeneration. We further propose the C57-*En1*^+/−^ model as a useful tool to assess the effect of non-genetic risk factors, and if such can override the genetic resistance to PD-like pathology in C57-*En1*^+/−^ mice.

In terms of strain differences, it is worth noting that at both 4 and 16 weeks of age, C57 and C57-*En1^+/−^* mice have lower counts of dopaminergic neurons in the SNpc compared to OF1 mice. This suggests that the observed resistance to the PD-like phenotype in C57-*En1^+/−^* mice is not due to a neuronal reserve in the SNpc exerting a buffering effect, but rather due to other mechanisms. Indeed, instead of SNpc cell numbers, cellular function and morphology may be more important for motor function, and the Collaborative Crossing Consortia have reported that low performance on motor tests is associated with striatal axonal branching rather than TH^+^ area in the SNpc (Thomas et al., 2021). Moreover, we detected no differences between C57 and OF1 in gene expression characteristic for mature dopaminergic neurons, suggesting that the difference in neuron number does not have an impact on relative transcript counts from dopaminergic neurons in this model. Since behavioral tests were not assessed in the current study, it remains to be answered how transcriptional profiles in *En1*-hemizygous mice are related to motor- and non-motor functions in C57 and OF1.

According to our results from analyzing gene expression in OF1-*En1*^+/−^ vs. OF1 1 week old mice, the neurodegenerative phenotype is preceded by lower expression of developmental genes. More specifically, DEGs were enriched for processes of nervous system development, including cell migration and axonal growth, which could reflect early changes associated with proper synapsis development. Particularly, semaphorin 3a (*Sma3a*) and plexins (*Plxna2* and *4*), that have been shown to be essential for neuronal development and axonal elongation, all had lower expression in OF1-*En1*^+/−^ compared to OF1 mice (Tamariz et al., 2010; Limoni and Niquille, 2021). Furthermore, transcriptomic studies in PD patients have reported an upregulation of the human orthologues to *Sma3a* and *Plxna4*, and rare variants in *PLXNA4* have been linked to PD (Schulte et al., 2013). This further reinforces that axon development and maintenance of axonal terminals are processes linked to PD and could be promising targets for modifying PD susceptibility (Tagliaferro and Burke, 2016; Berth and Lloyd, 2023).

When comparing transcriptomes between C57*-En1*^+/−^ and OF1*-En1*^+/−^ mice, we found a clear enrichment in OxPhos-related genes among the regulated transcripts, with consistent higher expression in C57-*En1*^+/−^. We also found a higher expression of OxPhos genes in C57-*En1*^+/−^ compared to C57 WT mice, suggesting compensatory mechanisms could underlie the resistance to progressive PD-like pathology in C57 mice. This is of particular interest, since administration of EN1 or EN2 protein to C57 WT mice has been found to increase complex I subunit proteins (Ndufs1 and Ndufs3) and protect midbrain dopaminergic neurons against complex I insults (Alvarez-Fischer et al., 2011; Rekaik et al., 2015b). Our results contribute new insights into the relation between *En*-genes and mitochondria, by showing that OxPhos gene expression can be induced under conditions of reduced *En1* expression and correlate with neuroprotection.

The consistent detection of differential gene expression of mitochondrial genes and genes that code for proteins in the OxPhos chain is likely to have functional impact, given the strong evidence linking mitochondrial impairment and energy metabolism to PD. This includes mitochondrial effects on progression and pathogenesis of PD models in different organisms (Briston and Hicks, 2018; Ordonez et al., 2018; Ikuno et al., 2021; Murali Mahadevan et al., 2021), the toxic effects of MPTP and Rotenone, a reduced activity of complex I in postmortem PD brains (Cappelletti et al., 2023) and the association between PD genes and mitochondria (Rekaik et al., 2015a). In addition, inhibition of the mitochondrial pyruvate carrier has been shown to directly slow down pyruvate oxidation in isolated brain mitochondria, to increase the utilization of other substrates, to normalize oxygen consumption after MPP+ treatment, to reduce dopaminergic neurodegeneration *in vitro* and *in vivo*, and well as to reduce motor impairment in OF1-*En1*^+/−^ mice (Ghosh et al., 2016).

Our results suggest that early mechanisms, before the onset of neurodegeneration, take place in the mitochondria and halt the neurodegenerative process in C57-*En1*^+/−^ mice up to 16 weeks of age. Downregulation of genes related to mitochondrial function, including OxPhos, was recently found to be consistent in brains with different Braak stages of PD pathology.

Further experiments are needed to quantify OxPhos proteins and to understand the effects that these gene expression changes may have on mitochondrial function, preferably with functional respirometry. In addition, the level of mitochondrial turnover and mitophagy could impact on dopaminergic cell metabolism and should be assessed.

Another potential neuroprotective effect could be mediated by lower expression of *Keap1* in C57-*En1*^+/−^ compared to C57 mice. The encoded Kelch-like ECH-associated protein 1 acts as a suppressor of *Nrf2,* which drives anti-inflammatory and antioxidative responses and is of high relevance to PD (Lin and Beal, 2006; Kopacz et al., 2020). The lower *Keap1* expression in C57-*En1*^+/−^ mice could thus disinhibit *Nrf2* and have neuroprotective effects. When comparing C57 and OF1 mice, processes related to T-cell mediated immunity, antigen presentation and toll-like receptor 4 signaling were significantly enriched. However, no clear pattern of differential expression of pro- or anti-inflammatory molecules was observed in any strain and highly polymorphic immune-related genes such as those encoding MHC molecules could have impacted on the mapping of transcripts to genes and influence strain comparisons.

This study has limitations, including the following considerations. We cannot rule out that the nigrostriatal pathology would evolve into dopaminergic neuron loss in the SNpc of C57-*En1*^+/−^ mice at an advanced age. Therefore, we cannot conclude if the neurodegenerative process is permanently halted or merely delayed, but both scenarios are of high relevance to PD, where both halted and delayed disease progression have significant clinical importance for most affected individuals. Additional studies of aged C57-*En1*^+/−^ mice are warranted to assess if neuroprotection is conserved over time. It is also worth considering that in order to avoid the higher intrastrain variation that we have previously observed with respect to neurodegeneration in *En1*^+/−^ female mice, this study was carried out only on male mice. The effects of OxPhos-related gene expression on PD-like phenotypes should thus be further investigated in both sexes. Another limitation of this work is that bulk-RNAseq does not address the contribution of different cell types. Despite being able to obtain samples that are highly enriched in dopaminergic neurons from the SNpc, we cannot disregard other cell types. Differences in sample cell composition have been shown to be a confounder in transcriptomic analyses performed in PD cohorts (Nido et al., 2020). However, in those cases, samples were dissected when the disease was advanced and therefore should lack many dopaminergic cells (Alves et al., 2009; Gaare et al., 2018). Single-cell RNAseq analyses would further contribute with cell-type specific transcriptome profiles as well as comparisons of cell type-specific composition between strains.

The results from this study provide further evidence on the importance of genetic background for the onset and progression of nigrostriatal degeneration. The data specifically suggest that differential expression of genes encoding mitochondrial proteins before the onset of dopaminergic neurodegeneration regulate the vulnerability of nigral dopaminergic neurons and susceptibility to PD. The fact that mitochondrial function and turnover are critical processes in PD is well known, but the findings presented here implies that normal genetic variation between strains, and potentially between humans, alters expression of genes encoding mitochondrial proteins. This opens for new therapeutic targets to prevent onset and/or progression of PD. In conclusion, this study shows that transcriptional changes linked to neuronal metabolism and mitochondrial physiology are key components of PD-related neurodegeneration and suggests that increased expression of genes encoding mitochondrial proteins have a key impact on resistance to disease onset and progression.

Most PD cases are idiopathic and caused by a complex interplay between genetic variants and environmental risk factors. However, the underlying mechanisms remain elusive. Here we show that *En1* hemizygosity leads to progressive nigrostriatal degeneration with a loss of dopaminergic neurons in OF1-*En1*^+/−^ but that C57-*En1*^+/−^ mice only exhibit early signs of nigrostriatal pathology and do not progress to a PD-like phenotype over time. We identified differences in gene expression related to oxidative phosphorylation before the onset of neurodegeneration to be associated with differential susceptibility to *En1*^+/−^ induced PD-like pathology. Our work shows how gene expression changes modulate vulnerability to dopaminergic neurodegeneration in the *En1*^+/−^ mouse and reveals putative molecular mechanisms behind the onset and progression of PD.

## Data availability statement

The datasets presented in this study can be found in online repositories. The names of the repository/repositories and accession number(s) can be found at: https://www.ncbi.nlm.nih.gov/geo/, GSE236461.

## Ethics statement

The animal study was approved by the local ethics committee Malmö/Lund region and specified if permit 4992/2022. The study was conducted in accordance with the local legislation and institutional requirements.

## Author contributions

LB: Data curation, Formal analysis, Funding acquisition, Methodology, Software, Visualization, Writing – original draft, Writing – review & editing. AD: Conceptualization, Data curation, Formal analysis, Investigation, Methodology, Software, Writing – review & editing. DR: Data curation, Formal analysis, Investigation, Software, Writing – review & editing. IJ-F: Conceptualization, Methodology, Writing – review & editing. FF: Data curation, Investigation, Writing – review & editing. SB: Investigation, Writing – review & editing. DA: Methodology Writing – review & editing. KAB: Data curation, Investigation, Software, Writing – review & editing. MS: Conceptualization, Funding acquisition, Project administration, Resources, Supervision, Writing – original draft, Writing – review & editing.

## Glossary

**Table 1:** 

Atp8	ATP synthase Fo subunit 8
C57	C57Bl/6j
DEGs	Differentially expressed genes
En1	Engrailed 1
En1^+/−^	Engrailed 1 hemizygosity
EN2	Engrailed 2
GO	Gene Ontology
GWAS	Genome-wide association studies
LCM	Laser-captured micro-dissected
LRRK2	Leucine-rich repeat kinase 2
mt	Mitochondrial
MHC	Major histocompatibility complex
NADH	Nicotinamide adenine dinucleotide
Nd	NADH–ubiquinone oxidoreductase chain 1
OF1	SwissOF1
O.N.	Over night
OxPhos	Oxidative phosphorylation
PD	Parkinson’s disease
PFA	Paraformaldehyde
PINK1	PTEN-induced kinase 1
Plxna	Plexin
QTLs	Quantitative Trait Loci
RIN	RNA integrity number
RNASeq	RNA Sequencing
ROI	Region of interest
Sma	Semaphorin
SNCA	Synuclein Alpha
SNpc	Substantia nigra pars compacta
TH	Tyrosine hydroxylase
VTA	Ventral tegmental area
WT	Wild-type

## Supplementary material

### Supplementary Table S1

List of differentially expressed genes.

### Supplementary Table S2

Results from enrichment analyses using Gene Ontology (GO), Kyoto Encyclopaedia of Genes and Genomes (KEGG), Reactome Pathway Analysis and Wikipathways.

### Supplementary FIGURE S1

Negative correlation between load of striatal axonal swellings and nigral dopaminergic cell counts in OF1-*En1*^+/-^ (A) but not in C57-*En1*^+/-^ mice (B). Load of axonal swellings; Tyrosine hydroxylase-positive (TH+) axonal swellings in the striatum relative to TH+ cell counts in SNpc at 16 weeks.

### Supplementary FIGURE S2

**(A)**. Principal component analysis (PCA) of the samples shows that the main effect is due to genetic background. **(B)**. Venn diagram illustrating the intersection between the four sets of differentially expressed genes (DEGs). **(C)**. Detected levels of *Engrailed 1 (En1)* in the RNA-Seq data. **(D)**. *En1* expression levels detected through RT-qPCR.
