# Supplementary figures and images for: Nigral transcriptomic profiles in Engrailed-1 hemizygous mouse models of Parkinson’s disease reveal upregulation of oxidative phosphorylation-related genes associated with delayed dopaminergic neurodegeneration
Source: Front Aging Neurosci. 2024 Feb 5;16:1337365. doi: 10.3389/fnagi.2024.1337365 (PMC10875038; doi:10.3389/fnagi.2024.1337365)

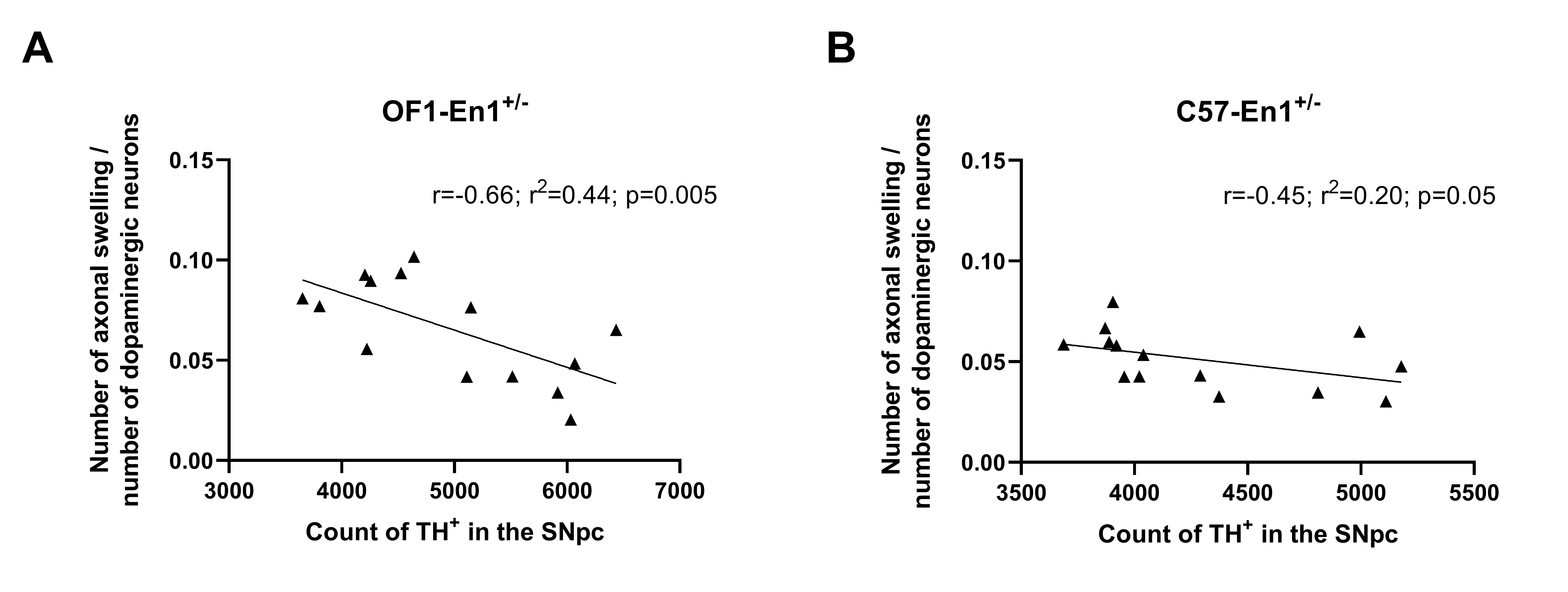

Supplement: Supplementary file 1 [file Image_1.TIF]

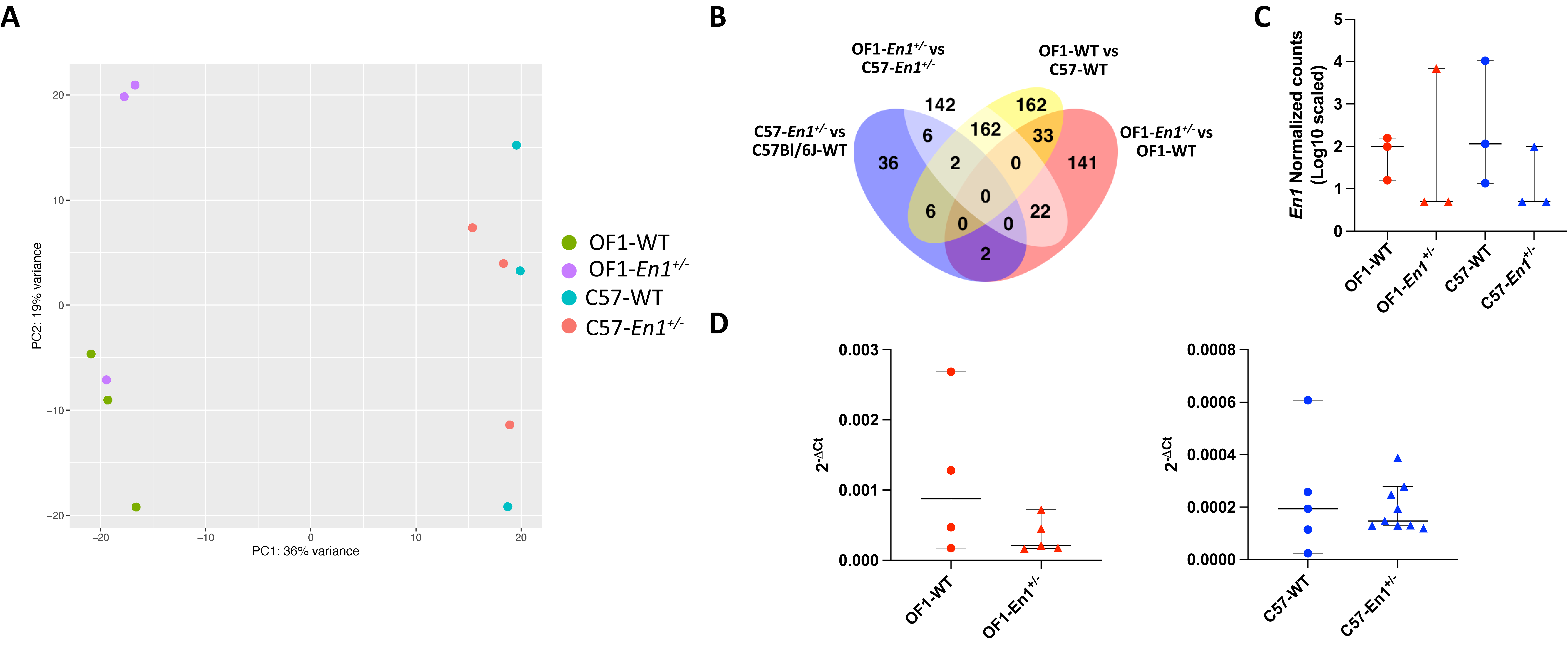

Supplement: Supplementary file 2 [file Image_2.TIF]
